# Understanding capability, opportunity, and motivation for at-home COVID-19 testing in underserved populations during the pandemic

**DOI:** 10.1093/tbm/ibag033

**Published:** 2026-06-11

**Authors:** Joni H Pierce, Peter Taber, Leticia Stevens, Adriana Rush, Ryzen Benson, Catherine Staes, Andy J King, Chelsey R Schlechter, Kimberly A Kaphingst, David W Wetter, Guilherme Del Fiol

**Affiliations:** Department of Biomedical Informatics, University of Utah, Salt Lake City, UT, United States; Department of Biomedical Informatics, University of Utah, Salt Lake City, UT, United States; Department of Biomedical Informatics, University of Utah, Salt Lake City, UT, United States; School of Medicine Greenville, University of South Carolina, Greenville, SC, United States; Department of Radiation Oncology and Bakar Computational Health Sciences Institute, University of California San Francisco, CA, United States; Department of Biomedical Informatics, University of Utah, Salt Lake City, UT, United States; Huntsman Cancer Institute, University of Utah, Salt Lake City, UT, United States; Department of Communication, University of Utah, Salt Lake City, UT, United States; Huntsman Cancer Institute, University of Utah, Salt Lake City, UT, United States; Department of Population Health Sciences, University of Utah, Salt Lake City, UT, United States; Huntsman Cancer Institute, University of Utah, Salt Lake City, UT, United States; Department of Communication, University of Utah, Salt Lake City, UT, United States; Huntsman Cancer Institute, University of Utah, Salt Lake City, UT, United States; Department of Population Health Sciences, University of Utah, Salt Lake City, UT, United States; Department of Biomedical Informatics, University of Utah, Salt Lake City, UT, United States

**Keywords:** patient decision-making, patient engagement, COVID-19 testing, COVID-19 prevention, information trust, chatbots

## Abstract

**Background and purpose:**

We investigated the capabilities, opportunities and motivations associated with decision making related to at-home COVID-19 testing in populations most affected by the pandemic.

**Methods:**

We conducted semi-structured interviews to explore the capability, opportunity and motivation for COVID-19 testing by the pandemic populations most affected by the pandemic. Interviews were carried out according to the Critical Incident Technique, and analysis and interpretation were guided by the COM-B model for behavior change.

**Results:**

We conducted 19 (13 in English, 6 in Spanish) semi-structured interviews with participants from both urban and rural areas in Utah. The following 6 key themes emerged: (i) Participants sought clear information about COVID-19 from trustworthy, credible sources, (ii) Economic concerns relating to job security and income loss as well as costs associated with COVID-19 testing were concerns, conversely access to free COVID-19 tests facilitated testing, (iii) Rapid testing with fast results at convenient locations including at-home were key facilitators to COVID-19 testing, (iv) Wide variation of trust levels related to at-home COVID-19 test accuracy, (v) Perception of risk to self and others, including misconceptions surrounding risk, and (vi) Intense emotions ranging from fear associated with loss to peace of mind associated with confirmation of a negative COVID-19 test.

**Conclusion:**

The six themes identified surfaced as facilitators, barriers, and key considerations depending upon the context when participants were engaging in decision-making about COVID-19 testing. Providing validated easy to understand information, from trustworthy sources, while addressing and minimizing concerns about economic implications associated with testing are critical to increase patient engagement.

## Teaser

Accurate and trustworthy health information and financial security are key concerns for patients most affected by the COVID-19 pandemic when considering testing at-home to detect and manage COVID-19 infection.

Implications **Practice:** The study findings suggest when designing public health interventions for COVID-19 and potentially other public health crises, providing validated easy to understand information from trustworthy sources might increase patient engagement with the intervention.

**Policy:** Developing strategies that work together to reduce barriers and enhance facilitators by addressing and minimizing the fears associated with a health intervention may also promote acceptance of the intervention and increase health equity.

**Research:** Future research should explore trust and key economic concerns to understand how known barriers that affect disproportionately affected groups can be mitigated with emphasis on methods to promote trust in information sources and delivery channels.

## Background and significance

While COVID-19 infection rates have declined across the U.S., COVID-19 has become an endemic disease like other seasonal viral infections [1, 2]. Since COVID-19 is a novel coronavirus with high adaptive mutation capacity, variants are expected to continue to mutate and evade previous immunity [1]. Furthermore, both natural and vaccine-induced immunity diminish over time moving individuals from protected to vulnerable status [3–5]. As the future course of COVID-19 evolves, testing and vaccination for COVID-19 will continue to be key prevention, management, and containment strategies.

### Disparities in the COVID-19 pandemic

During the COVID-19 pandemic, individuals with low socioeconomic status (SES) and from rural areas in the United States experienced disproportionate negative health impacts due to COVID-19 [6, 7], including a 36.9% higher cumulative mortality rate in rural areas (383.5 per 100 000) relative to urban areas (280.1 per 100 000) [8]. COVID-19 related health inequities are magnified in disproportionately affected groups due to fear and misinformation associated both with the disease itself and with its prevention methods [9]. One reason for disparities is a lack of access to credible COVID-19 information and a lack of awareness of and access to COVID-19 testing resources [10, 11]. Multiple converging factors amplify health disparities associated with COVID-19 in rural communities [12–14]. The use of at-home COVID-19 tests is an important tool in the ongoing public health response against COVID-19 [15]. Previous research shows that at home COVID-19 testing is performed most often by people who are white, educated and in upper income levels (e.g. >$150 000 per year) [16]. This highlights the ongoing need for interventions to reduce disparities in COVID-19 testing among populations who are at a higher risk of adverse COVID-19 outcomes. Importantly, the availability of low or no cost at home COVID-19 tests is a key strategy to promote health equity.

### Digital health interventions

According to a 2025 survey by Pew Research, 82% of people earning $30 000/year or less own a smartphone [17]. As such, digital health interventions, such as text messaging and conversational agents (i.e. chatbots), are promising strategies to increase access to at-home COVID-19 testing among populations facing health disparities. Chatbots are computer programs designed to simulate human conversation [18, 19]. Chatbots can be accessed on devices such as smartphones, and desktop or laptop computers [18]. Systematic reviews have shown that interventions using chatbots are effective for a wide range of clinical problems, including treatment and monitoring, health services support, diagnostics, education, and lifestyle and behavioral change [20]. Chatbots can reach users where health care is limited and offer a range of benefits such as convenience, scalability, personalization, and ease of use controlled by the patient [21]. We found several design studies for the development of chatbots aimed at underserved populations. However, we were unable to find any studies in the published literature exploring the decision-making processes around COVID-19 testing for underserved populations who engaged with a chatbot [22–25].

## Purpose

We investigated the capabilities, opportunities and motivations associated with decision-making related to at-home COVID-19 testing in populations most affected by the pandemic. To accomplish this, we researched the psychological and physical capacity, social and environmental factors, and roles, beliefs, goals and emotions that drive COVID-19 testing behavior. These findings informed the development of conversational scripts for a smartphone-based chatbot intervention. This intervention was designed to promote at-home COVID-19 testing in the SCALE-UP II pragmatic clinical trial [26] (trial registration: clinicaltrials.gov NCT05533918 and NCT05533359).

## Materials and methods

### Design

We performed an exploratory qualitative study using in-depth semi-structured interviews via Web teleconference to explore the capability, opportunity and motivation for COVID-19 testing among populations most affected by the pandemic. The University of Utah Institutional Review Board under protocol number IRB_001156 approved the study. The researchers obtained informed consent from all participants. We anonymized all transcript data.

### Participants and setting

The study population included adults aged 18+ years in rural or urban areas in Utah who spoke English or Spanish. Participants were required to meet at least one of the following criteria: had no college degree, received primary care at a safety net community health clinic or free clinic, had no health insurance or had Medicaid insurance, or reported housing insecurity or food insecurity. We assessed food and housing insecurity through a screening questionnaire where participants were asked how often they worried or stressed about having enough money for food or rent/mortgage. We considered those who responded “always” or “usually” to be eligible for the study (see Appendix 1 for the full study eligibility criteria). Rurality was determined based on the participants’ zip code and according to the Federal Office of Rural Health Policy (FORHP) under the Health Resources & Services Administration (HRSA) [27].

### Recruitment/participant identification process

We aimed to recruit up to 30 participants including 20 English-speaking participants, 10 Spanish speaking, divided evenly between urban and rural. A mix of passive and active recruiting methods was used to recruit participants [28]. Active methods included targeting specific clinics and individuals for recruiting, whereas passive methods include all other forms or recruitment such as websites and posters. Recruitment strategies included: (i) attending a community health fair along with the University of Utah Community Collaboration and Engagement Team (CCET) to raise awareness about this study, (ii) placing an advertisement at the Utah Rural Health Association social media pages, (iii) working with Utah Community Health Center (CHC) leaders in both urban and rural settings to raise awareness through posters placed at the clinics, (iv) posting digital advertisements in social media, (v) working with the CCET on outreach to the targeted population through online advertising, email communication to clinics and community health events [29], and (vi) publishing a study webpage through the University of Utah study locator website.

The study team deployed broad recruitment methods to recruit participants including both targeted recruiting through outreach to clinics and passive recruiting through social media advertising and a study locator website. Those efforts resulted in 262 individuals who indicated interest in participating in the study and were asked to complete a REDCap survey to verify study eligibility. Recruitment continued until we achieved saturation as noted by the absence of information that would result in new themes.

### Conceptual framework

The COM-B model guided the analysis and interpretation of the interview data [30]. The COM-B model describes three core domains that drive behavior change including capability, opportunity and motivation. Capability includes psychological and physical capacity associated with the person; opportunity includes social and environmental factors; and motivation includes roles, beliefs, goals and emotions that drive behavior.

The COM-B model identifies three factors that need to be present and interact in a dynamic way for behavior to occur. Specifically, capability and opportunity must be present to support motivation, which has a direct relationship with behavior. Within the COM-B model, motivation is the precursor for behavior, but capability and opportunity are not directly related as prompts for behavior. These factors support motivation, which can directly influence behavioral actions. Within the COM-B model, motivation is the central tenant, however without opportunity and capability, motivation alone will not cause behavior to occur. According to West and Michie, within the human motivational system, wants and needs are generated by processes involving associations with positive and negative emotions. Thus, within the context of the COM-B model we see a dynamic system of both positive and negative feedback loops [31, 32].

**Figure 1:**
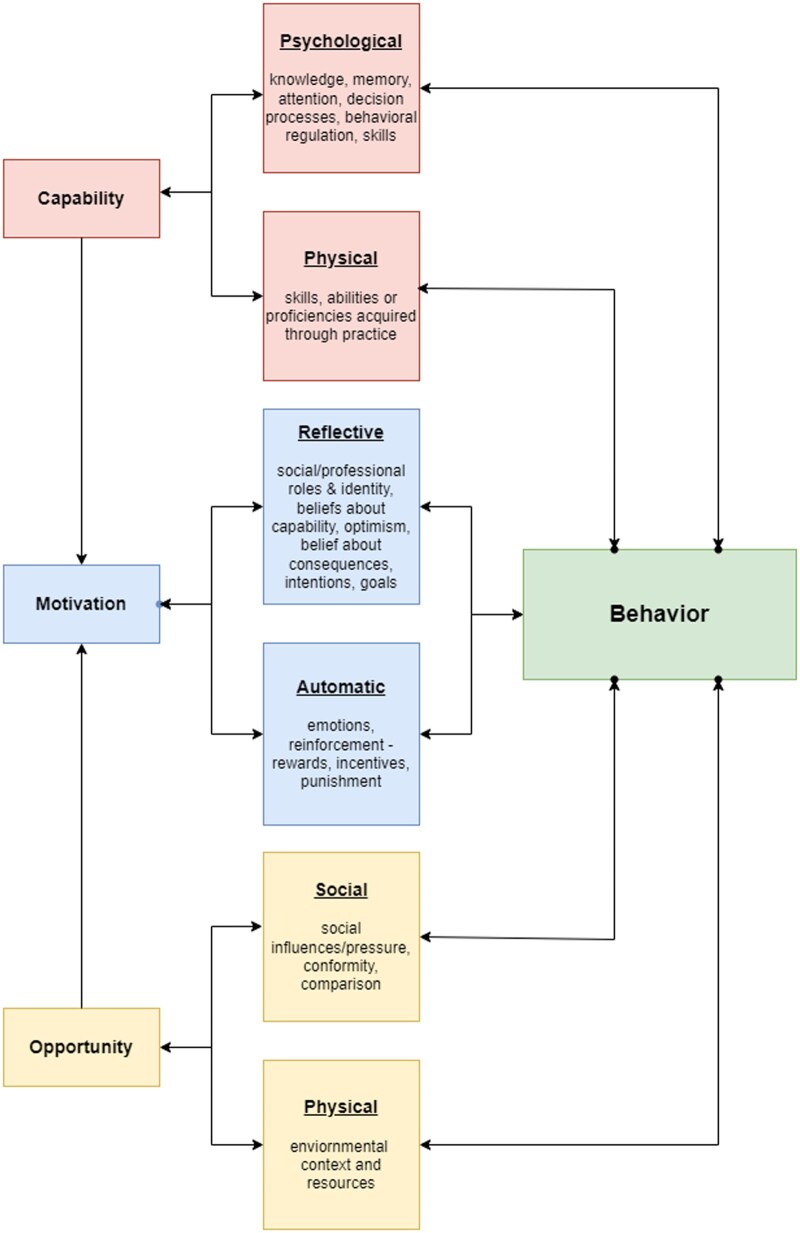
COM-B model for behavior change [30]. Depicts the relationship between capabilities, opportunities and motivators and the subcomponents that precede motivation to prompts behavioral action (Michie *et al*. [30]).

We chose the COM-B model because it is a framework used in health behavior research due to its explanatory value related to the psychological and environmental enabling factors necessary to support an individual in health behavior change (Fig. 1) [30].

### Procedures

The research team conducted individual interviews via Web conference between July 2022 and January 2023 through a HIPAA compliant version of Zoom (San Jose, CA, United States). Interviews were 20–30 minutes in length, and we offered compensation for participation.

### Critical incident technique

We used the Critical Incident Technique (CIT) to guide the semi-structured interview processes [33–36]. CIT is a qualitative interview method used for understanding human behavior in defined situations. CIT focuses on critical “incidents” to elicit effective and ineffective practices. A critical incident is defined as an event where upon the person involved is able to make a judgment of the positive or negative impact the incident had on the outcome of the situation [37]. We selected the CIT for this study because this method has been widely used in healthcare research and general qualitative research, and is highly flexible, yet effective at stimulating detailed recall about specific incidents. Normative responses, which can be influenced by social expectations, may be mitigated through questions that are directed to a specific event. Additionally, CIT helps interviewees focus on the concrete details of specific scenarios and avoid providing generic or highly scripted descriptions of their activities [35]. We created an interview guide with questions based on the Critical Decision Method (CDM), which is a form of the CIT (see Online Supplement—CA Design Study Interview Guide v9). The guide was piloted with four researchers and refined based on pilot findings [33].

### Semi-structured interview processes

We used a four-step process to uncover capabilities, opportunities and motivations when making critical decisions focused on COVID-19 testing.

In the first step, we asked participants to recall and briefly describe an actual incident where they had to decide whether to pursue COVID-19 testing. Second, we asked participants to draw a linear timeline showing key points before and after the COVID-19 testing decision point. Participants were then asked to describe in detail their thinking and considerations prior to and following the decision about COVID-19 testing. Third, using the timeline, we presented probing questions to participants to explore the COVID-19 testing decision incident along with all considerations in more detail. Last, we posed “what if” scenarios to the participants through hypothetical questions to stimulate additional memories and potential solutions (Online Supplement—CA Design Study Interview Guide v9) [38, 39]. The study team recorded participant interviews in a HIPAA compliant environment and then had the audio interviews professionally transcribed. All Spanish audio interviews were first translated to English and then transcribed into text. All data collected during the study was stored on a secure server at the University of Utah.

### Analysis

Thematic analysis was performed following procedures described by Crabtree and Miller [40]. A team of three researchers met weekly to discuss, unitize, analyze and code interview transcripts. The team conducted multiple iterations of transcript review, discussion, and coding during the weekly meetings. Members of the research team performed independent coding for three to four transcripts with comparative evaluation to resolve any discrepancies. The research team continued to meet regularly to iteratively code all unitized topics. The study team agreed upon final coding for transcript sections through discussion, debate and consensus. We analyzed transcripts using Dedoose coding software (SocioCultural Research Consultants, LLC, Los Angeles, CA). Next, the research team together mapped the codes to the COM-B model for thematic analysis and interpretation. The research team followed these steps:

*Step 1: Sampling*. We collected data through semi-structured interviews with audio recordings, followed by professional transcription into text.

*Step 2: Describing/Unitizing*. The research team unitized text segments from the interviews by discussing and deciding what comprises a discrete unit. A unit typically began with a new topic and ended when a new topic was introduced to the conversation.

*Step 3: Organizing.* The study team organized interviews into topics for coding. We created codes by using a hybrid deductive and inductive process and by starting with a preliminary set of coding tags. During this process, we coded for common groups that aligned under the following main categories: (i) key considerations and decision points for COVID-19 testing, (ii) barriers to COVID-19 testing, and (iii) facilitators for COVID-19 testing.

*Step 4: Connecting.* We iteratively refined and expanded codes and sub-codes using an inductive coding approach.

*Step 5: Corroborating.* The research team validated codes and coded text by determining a shared understanding of what the codes meant and how we would apply them to the text. We developed additional codes, edits, and consolidations to the predetermined code set as we continued analysis of the transcripts. Disagreements were resolved through discussion and consensus. Following this, JP and LS divided the remaining transcripts and coded them independently.

*Step 6: Thematic analysis & COM-B mapping.* The research team met to derive themes from the coded data clusters through debate, deliberation and consensus. We referenced the COM-B model for behavioral change in order to group codes into a framework for interpretation.

We followed the Standards for Reporting Qualitative Research (SRQR) standard for qualitative reporting [41].

## Results

### Interview results

We screened 252 potential participants using a REDCap survey (Appendix 1). The majority of participants were Female 16 (84%), Non-Hispanic/Latino 12 (63%), lived in urban areas 17 (89%), and spoke English 13 (68%) Table 1.

**Table 1:** Study population demographics (Aug 2022–April 2023).^a^

	All *N* = 19	English language *n* = 13	Spanish language *n* = 6
Sex			
Male	3 (16%)	3 (23%)	0 (0%)
Female	16 (84%)	10 (77%)	6 (100%)
Ethnicity			
Not Hispanic/Latino	12 (63%)	12 (92%)	0 (0%)
Hispanic/Latino	7 (37%)	1 (8%)	6 (100%)
Geographic (residence)			
Urban	17 (89%)	11 (85%)	6 (100%)
Rural	2 (11%)	2 (15%)	0 (0%)

a A qualitative study using the Critical Incident Technique (CIT) for interviews, with interview data mapped to COM-B model of behavior change for categorization and interpretation to understand the decision-making factors related to COVID-19 testing among patients who receive care at low resource community health centers (CHCs) across the state of Utah.

We identified six key themes and mapped the key themes in the following four COM-B subcategories: psychological capability, physical opportunity, reflective motivation and autonomic motivation (Table 2).

**Table 2:** Key themes mapped to COM-B model for behavior change along with exemplar participant quotations (Aug 2022–April 2023).^a^

COM-B subcomponent	Theme	Sample quotations
Psychological capability- (knowledge)	Theme 1. Clear information about COVID-19 from credible sources made testing more attractive.	*“It was trying to navigate the uncertainty introduced by—well, there was a lot of uncertainty introduced by COVID, like whether or not the tests were correct and then the CDC information about how long you’re contagious for, and what is actually safe or not, and so there was a lot to try to navigate.” English Urban P6* *“If I would test positive, what would I like to know? It’d be the symptoms that I should look for. I do like step-by-step guides of what you should.” (English Urban P2)* *“I asked the doctor, ‘At what point do I need to go to a hospital, or start worrying a lot?' He told me, 'When you can’t breathe’ I didn’t get that bad, but it got worse.” Spanish Urban P1* *“I don’t like to watch the news nowadays. I don’t trust what I see on the internet, either. If I go to my doctor I trust him or her and if he or she is recommending them it is for my own wellbeing and that of my family.” Spanish Urban P2* *“I have no idea, myself, because the information that’s been given is so mixed. The people who talk on TV say one thing and then the CDC, I sometimes get on the—when I go on Facebook, something about it, and I do read it to see ‘cause I’m nosy, what it’s saying, but it is real, I can’t just consume myself in it’ cause then it makes me have fear.” English Urban P12*
Physical opportunity (resources)	Theme 2. Economic concerns strongly influenced testing decisions, including job security and loss of income as well as costs associated with COVID-19 testing. Theme 3. Rapid testing with fast results at convenient locations including at-home was a key facilitator of COVID-19 testing.	*“I know I would get time off if I did happen to get COVID. That really helps knowing that there’s not any financial consequences.” English Urban P3* *“I think my main concern would have been—yeah. It’s tough because I know that at that point I would have needed to cancel my trip and that would have been really, really bad for my career. That’s one of the reasons why I didn’t do the PCR.” English Urban P5* *“My husband works and is the main provider of the home, so if he got sick, we would have financial difficulties. That would be my concern if we got sick.” Spanish Urban P5* *“If I had to pay for it, I wouldn’t be taking it as often. Then I would only do it if necessary at that point.” English Urban P5* *“I would say I would use the free ones at home. I would try to avoid paying anything. If it came to that and I had to pay $20.00, I don’t think it’s that bad, but I would rather not.” English Urban P10* *“I thought it was really great, and I don’t know if a lot of people knew that you could contact the US, the mail, and get it sent to you through the mail. I was able get some home tests for free through the mail and stuff like that. I honestly would not have tested near as often if it was not free if it wasn’t that availability.” English Rural P13* *“To go to a designated place is very tedious because you have to stand in very big lines; you have to be there for a long time. On the other hand, I like more the home kits because if you’re feeling a little ill, you can do it yourself on your own and you get the results in 15 to 20 minutes. It’s more convenient and you don’t get exposed that much by going there; I like the home ones better.” Spanish Urban P1* *“I didn’t have to leave the house. I could do it from home. Also, I did it at night when my children were asleep. I was alone and relaxed, so I could do it. English Urban P10* *“I did the quick little at home test that I had gotten from the government in the mail and just followed the instructions. It was an easy test.” English Urban P11* *“I thought it was pretty straightforward. It was convenient having it here. I didn’t have to drive—I live in a rural area. I would have had to have driven 20 minutes to get to the nearest.” English Rural P11*
Reflective motivation (beliefs, optimism)	Theme 4. Trust level for at-home COVID-19 test accuracy varied widely. Theme 5. Risk of exposure and disease for oneself and others influenced decisions to test.	*“I don’t trust the home tests anymore. After I came to know, after I spoke to other people who tested positive and they told me that it doesn’t. Even though the PCR came positive, the home tests kept showing it’s negative. After that, now I no longer trust the home tests.” English Urban 5* *“I did not rely on it as much because there are high chances of getting false negative results…It’s not positive, but again, I think that if anybody has symptoms, they should not just rely on the home test.“ English Urban 7* *“I would put it at probably a 90% trust level. There was times in the past where I would do a home-based one, and it would come back negative.” English Rural 13* *“Yeah, I trust them 'cause most of the tests have a pretty high accuracy percentage. There’s gonna be some cases where there’s false positives or negatives, but I think definitely it’s pretty accurate. I don’t know. If I felt like the test was really off, I could always retest.” English Urban P3* *“Yes, I was confident because I didn’t have the symptoms, so I believed the results.” English Urban P8* *“Whenever I’ve decided about whether or not to get tested, I usually try to weigh out what I would be sacrificing by getting tested or what level of potential exposure I think I have. If I didn’t have any symptoms since I’m vaccinated, especially, I wouldn’t be as motivated to get tested now.” English Urban P3* *“If the symptoms were really bad, I would do something, but then now they say the symptoms are not bad, and then there’s the asymptomatic people that are just spreading, and then I thought, well, am I one of those?” English Urban P12* *“I have family members that are high risk for COVID with breathing, asthma, different things like that. I always make sure that I was doing what I needed to make sure that I was being safe so I could go around my family, and be able to go to work and not spread it or contract it, or anything like that.” English Rural P13*
Automatic motivation (feelings/emotions)	Theme 6. Emotional responses to testing and the disease included fear of loss and peace of mind associated with confirmation of a negative COVID-19 test.	*“At first, I was feeling, yeah, skeptical about having this test, and that was because I—at first, I was thinking maybe there was something attached to it. The day before the COVID test, I was just having the—this feeling of it having a negative effect in the future, I mean, affecting our life in the future, so we were just feeling scared.” English Urban P9* *“I have friends who tell me that the little testings have COVID on 'em, so if you test for it, you’re gonna get it anyway. I know that’s ludicrous, but oh.” English Urban P12* *“It seems like a lot of our elders and things like that tend to get COVID a lot more frequently and get worse cases and things like that down on the reservation. It just was that relief and knowledge knowing that all of us are clear bill of health and that we can go down and now enjoy our time and know that we’re not putting anyone at risk to be sick. It’s just that relief knowing that we’re not gonna infect anybody, and hopefully we don’t bring it back with us type thing and continue to spread it around.” English Urban P13*

a A qualitative study using the Critical Incident Technique (CIT) for interviews, with interview data mapped to COM-B Model of behavior change for categorization and interpretation to understand the decision-making factors related to COVID-19 testing among patients who receive care at low resource community health centers (CHCs) across the state of Utah.

#### Psychological capability

##### Theme 1. Clear information about COVID-19 from credible sources made testing more attractive

Participants identified a range of knowledge and information needs and indicated a lack of knowledge about COVID-19 infection symptoms and management. For example, they wanted to know about the relationship between exposure to COVID-19 and the elapsed time before it can be detected by an at-home COVID-19 test. Furthermore, participants inquired about testing procedures and discomfort associated with testing. Overall, participants reported a need for more information in several areas related to COVID-19 including prevention, infection, testing, and treatment. Participants reported mixed messaging in public health information found in media, online sources, and with authoritative agencies, which contributed to confusion and distrust.

#### Physical opportunity

##### Theme 2. Economic concerns strongly influenced testing decisions, including job security and loss of income as well as costs associated with COVID-19 testing

Participants described concerns related to a loss of income if they were to test positive or become sick with COVID-19 and had to quarantine. Often, these participants did not have insurance or paid time off benefits from their jobs and so they reported that they would go to work while sick and attempt social distancing. A reported obstacle to testing was having a documented positive test that could affect immediate career opportunities.

Participants reported access to free at-home COVID-19 tests as a facilitator for more frequent testing. Participants reported that having to assume financial costs associated with COVID-19 tests would result in a decision not to test in some cases. With more frequent testing, participants reported gaining peace of mind through testing before events and gatherings.

##### Theme 3. Rapid testing with fast results at convenient locations including at-home was a key facilitator of COVID-19 testing

The participants described the ability to test quickly on-demand at home as a facilitator of COVID-19 testing. Participants were very positive about the speed and convenience of testing at home and associated this level of access as making the testing process “easy”. In contrast, participants noted long lines at clinics and in some cases inconvenient times and locations to obtain COVID-19 tests conducted by others.

#### Reflective motivation

##### Theme 4. Trust level for at-home COVID-19 test accuracy varied widely

Participants reported some hesitation in trusting test results, especially if they were negative but symptoms were present. Across the interviews, there was wide variation in the accuracy of beliefs about self-administered COVID-19 tests and the meaning of results. Some participants had a clear understanding of the possibility of asymptomatic infection while others reported the lack of symptoms as evidence for no COVID-19 infection. Trust in test accuracy ranged from trust with some limitations associated with at-home test kit performance to a lack of trust in the test due to false negative/positive results.

##### Theme 5. Risk of exposure and disease for oneself and others influenced decisions to test

Some participants reported lack of symptoms and prior vaccination as reasons not to test for COVID-19. Participants with a history of COVID-19 infection or vaccination believed testing was not beneficial. It appears these participants may have had misconceptions about their personal risk for COVID-19 and had overconfidence in the durability of personal immunity status.

Several participants indicated a high level of concern about risk for others, especially those at risk as defined by age, pregnancy and health vulnerabilities. Many of the participants interviewed expressed a sense of responsibility and positive intentions in protecting others from COVID-19, which appeared to supersede their risk concerns for self.

#### Automatic motivation

##### Theme 6. Emotional responses to testing and the disease included fear of loss and peace of mind associated with confirmation of a negative COVID-19 test

Participants described fear as a concern related to painful or uncomfortable testing due to deep nasal swabs, which prompted avoidance of COVID-19 testing. They also described fear with relation to a distrust of COVID-19 test kits. Personal experience and experiences shared by others preceded these fears. Participants also described fear due to suspicion of potential intentional test kit contamination. In contrast, participants described emotions associated with peace of mind in relation to the protection of others as a motivating factor to obtain and use at-home COVID-19 tests.

## Discussion

Patients seek easy to understand information from trustworthy sources, which includes a patient’s personal clinician. Some participants described government sources as untrustworthy, especially historically marginalized people. Practical economic considerations are a key driver for decision making related to COVID-19 testing. Specifically, patients under economic pressure state that they do not have the option of taking unpaid time off work.

Through CIT interviews, analyzed and interpreted with the COM-B model, we were able to identify barriers, facilitators and considerations associated with decision making about at-home COVID-19 testing in populations most affected by the pandemic, such as racial and ethnic minorities, and those with low socioeconomic status primarily in urban settings. Four COM-B model subcategories emerged in this study: psychological capability, physical opportunity, reflective motivation and autonomic motivation. This study contributes to the existing literature by presenting the findings interpreted through the COM-B model for behavior change. In particular, trust surfaced as a common and prominent concern across several themes. Consequently, trust should be carefully considered when establishing information resources and associated delivery channels, developing educational content, addressing testing accuracy, and mitigating financial considerations. This is an important step to advance health equity for disproportionately affected populations,

### Theme 1. Clear information about COVID-19 from credible sources made testing more attractive

Our study highlighted the need for credible and consistent information as participants reported uncertainty and lack of trust in public sources of information due to mixed messages. This is consistent with previous studies on both general populations and disproportionately affected groups [42–46]. Several studies reported that community organizations including faith-based organizations, clinics, and health professionals, were considered to be trusted sources by individuals from disproportionately affected groups [44, 45, 47]. Public health interventions involving chatbots should leverage these trusted resources to disseminate information and provide access to COVID-19 testing to disproportionately affected communities to increase trust and potentially reduce health inequities.

### Theme 2. Economic concerns strongly influenced testing decisions, including job security and loss of income as well as costs associated with COVID-19 testing

Participants indicated having to make tradeoffs between following health guidelines and maintaining income sources. This is consistent with the existing published literature [48, 49]. Butler et al. studied concerns among Black/African American, Latinx, and Chinese American individuals associated with COVID-19 and found food and housing insecurity due to job loss was a major concern [44]. In a study of Latinx and Indigenous Latin American immigrant communities, Gehlbach at al. noted high levels of fear that COVID-19 testing could lead to potential loss of income [50]. The implications of our study along with the findings in the literature suggest the cost of testing is a major obstacle to COVID-19 testing. Future research could focus on interventions at the patient motivation level and policy changes focused on employment requirements. Providing no cost at-home COVID tests is an essential public health strategy to remove cost barriers and associated economic impact of testing. Public health professions could reduce isolation time to encourage isolation in the early stages of the disease when transmissibility is highest.

### Theme 3. Rapid testing with fast results at convenient locations including at-home was a key facilitator of COVID-19 testing

Our study findings were consistent with previous studies focused on obstacles to COVID-19 testing in disproportionately affected groups. In a study by McElfish et al., participants reported having to wait for up to two weeks to obtain test results, which is not practical for measures such as decisions regarding quarantine and seeking timely treatment [46]. Other studies reported accessible testing and ease of use as key facilitators to promote COVID-19 testing [45, 51]. Future public health interventions should focus on optimizing locational convenience and speed for testing. Home-based testing along with digital health tools for diseases and conditions including COVID-19 may be one of several modalities to address and mitigate large-scale public health events.

### Theme 4. Trust level for at-home COVID-19 test accuracy varied widely

Many participants in our study reported trust in the results of at-home COVID-19 tests with some caveats, which was consistent with the existing literature [52]. Two studies noted that participants who used at-home COVID-19 tests were less likely to isolate than those who received provider-administered tests [53, 54]. Moonan et al. also reported some negative points associated with at-home testing, including lost opportunities for providers to offer patient education, reinforce evolving recommendations, and emphasize mitigation behavior [54]. The implications of these findings underscore the need to continue to educate patients about the sensitivity of at-home tests along with instructions about how to reduce misinterpretation of test findings. Participants in some studies indicated a need for a central source for credible information while at the same time indicating a high level of distrust for government organizations [55, 56]. This paradox underscores the need for centralized information from sources deemed trustworthy by disproportionately affected communities.

### Theme 5. Risk of exposure and disease for oneself and others influenced decisions to test

Overt distrust also surfaced as participants described fear associated with beliefs from individuals in their social communities that free test kit suppliers may have intentionally contaminated test kits with COVID-19. Several previous studies reported deep mistrust of healthcare and government organizations, along with misconceptions stemming from personal contacts [44]. [45, 47, 50, 57, 58]. Further studies should be undertaken to understand how disproportionately affected communities determine credibility for information sources, specifically what attributes bolster perceived credibility. Those findings should be incorporated into targeted patient education information offered through specific venues considered credible and trustworthy by disproportionately affected communities.

### Theme 6. Emotional responses to testing and the disease included fear of loss and peace of mind associated with confirmation of a negative COVID-19 test

Prior studies have shown fear of mortality due to COVID-19, loss of job and income, social stigma and fear of pain from testing procedures [46, 58–60]. Consistent with the literature, fear was the most prominent emotion reported in our study. Fear can function as a motivator or an inhibitor to testing depending upon the context. For example, participants reported fear of painful testing associated with deep nasal swabbing, which was a significant obstacle to testing. Additionally, fear of job or income loss due to COVID-19 infection created an obstacle to testing. In contrast, fear of the health consequences of COVID-19 or transmitting COVID-19 to others served as motivation for testing as a contributor to “peace of mind”, which is consistent with findings in previous studies [61–63]. This was associated with safety for self and others, especially vulnerable people. This indicates that health communication messages with personal responsibility appeals may motivate COVID-19 testing in some participants.

## Limitations

This study had some limitations. First, the study participants only included individuals who live in Utah. Furthermore, the study population contained a high percentage of female urban participants, which may limit generalizability to male and rural populations. Nevertheless, the study findings are generally consistent with prior literature and may still be generalizable to similar populations in other US states. Second, we collected the interview data in 2022 when confusing and conflicting COVID-19 information may have saturated the public news venues, thus study findings may not generalize to earlier stages of a pandemic. Last, since the COVID-19 pandemic became highly politicized and plagued by mis/dis/mal information consequently, it is possible that the study findings will not generalize to other public health contexts [64–66].

## Conclusions

The study findings suggest when designing public health interventions for COVID-19 and potentially other public health crises, providing validated easy to understand information from trustworthy sources might increase patient engagement with the intervention. This may suggest that widespread government education programs may face challenges as a perceived trustworthy source of information. Our research findings were consistent with prior research, which suggests that underserved populations consider personal clinicians to be reliable sources of information. Consequently, offering education by clinicians at the point of care and on clinic websites and patient portal may prove to be more effective than large institution-based awareness campaigns. Addressing and minimizing fear associated with an intervention may also increase acceptance of the intervention. Further research should explore the viability of implementing chatbot interventions for other public health priorities. Additionally, new research should explore methods to promote trust in information sources and delivery channels. Key economic concerns should be studied further to understand how known barriers for disproportionately affected groups could be mitigated. The above strategies can work together to reduce barriers and enhance facilitators to support positive health behaviors, which may increase health equity.

## Supplementary Material

ibag033_Supplementary_Data

## Welfare of animals

This article does not contain any studies with animals performed by any of the authors.

## Appendix 1

### Chatbot study inclusion screening questions

1. Are you younger than 18 years of age? Yes > exclude and STOP
2. Are you currently in college or have you completed a college degree? Yes > exclude and STOP
3. Where do you get your primary healthcare?
  - CHC or free clinic > Include and STOP
4. Do you have health insurance?
  - No > include and STOP
  - Yes
    - Is it Medicaid?
      1. Yes > include and STOP
5. How often in the past 12 months would you say you were worried or stressed about having enough money to pay your rent or mortgage?
  - Always/usually > Include and STOP
6. How often in the past 12 months would you say you were worried or stressed about having enough money to buy nutritious meals?
  - Always/usually > Include and STOP
  - Rarely/Never > exclude and STOP

### Urban/rural classification

What is your zip code?
  - Classify for rural or urban
    - 15 urban > STOP
    - 15 rural > STOP
