# Supplementary material for: Understanding capability, opportunity, and motivation for at-home COVID-19 testing in underserved populations during the pandemic
Source: Transl Behav Med. 2026 Jun 11;16(1):ibag033. doi: 10.1093/tbm/ibag033 (PMC13256029; doi:10.1093/tbm/ibag033)
Supplement: ibag033_Supplementary_Data [file ibag033_supplementary_data.docx]

## **Interview Guide using Critical Incident Technique**

## **Conversational Agent Design Study**

Phase 1 – 1:1 Semi-Structured Interviews

## Outcomes

## COVID testing critical Incident stories for each person which can include one affirmative and declining decision, however it is also ok to accept stories that describe struggling with testing decisions regardless of outcome.

## Note: This is a sample guide; the actual wording or topics covered may be adjusted

Items Needed:

1. Consent cover letter
2. Copy of this script
3. Demographic data collection sheet
4. Paper and pens/pencils for participant to draw timeline of events
5. Smartphone or computer to access rural health screening website (fully charged)
6. Printed zip codes for rural health locations
7. Two recording methods (fully charged)
   1. If collecting data at an outside community event (use 1 for the researcher and 1 for the participant)
   2. If collecting data on the phone/Zoom/quiet room (use 1 to capture the entire interview and 1 as a backup)
8. Flyer to promote study (in person events only)
9. Take away small flyers to promote study with others (in person events only).
10. Notebook with participant ID codes and email addresses for gift cards.
11. Physical gift cards if available (in person only)
12. Focus group permission to contact list
13. Gift card email list

| **Steps** | **Sample Verbiage** |
| --- | --- |
| Introduction | “Hello, thank you for participating in this study. Your input is very valuable and will help us create new tools that are helpful when making decisions about COVID testing.”  “Today I would like to learn about your thoughts, feelings, and experiences when making decisions about COVID-19 testing.”  “I will be asking you to tell me about two times when you made a decision about COVID testing. The purpose of this is to help researchers understand how a phone based tool can help support people here in Utah when making decisions about COVID testing. Any information you provide will be helpful.”  “There are no right or wrong answers to these questions and different people can have different thoughts and experiences.“  “I am interested in hearing what you think, so my role will be to ask questions and listen to your responses.”  “This session will last about 30 minutes.” |
| Review consent letter | “You do not have to choose to participate in this study. Whether or not you participate will not affect your health care.”  “Please remember that you may choose not to answer any question and you may stop participating at any time.”  <If cover letter sent previously>   - “Did you have an opportunity to review the cover letter?” - “Do you have any questions?”   <If participant has not reviewed the cover letter – show it on Zoom or show a paper copy if live>  “Here is the cover letter that describes the study and your rights as a participant.“   - “Please review the cover letter”   <researcher read this verbatim - key points of the cover letter>  “The purpose of this study is to learn about the experiences of different people when making decisions about Covid-19 testing. The goal is to understand how an interactive tool may be helpful to you when making decisions about Covid-19 testing. We will ask you for your thoughts about whether such a tool would be helpful to you.  We will ask you about your thoughts and experiences about using health tools on your phone. We will record these sessions so that we have your thoughts in your own words. All recordings are kept confidential and only used for research purposes. This session is expected to be a 30 minute interview where you will be asked about your experiences when making decisions about Covid-19 testing.  You would receive a $50 gift card for this session which will be sent to you by email.  We may use your words in research publications or presentations. However, your answers will not be presented with your name or other information that can identify you.  It is possible that someone could see your information as a result of taking part in this study. However, we take a number of steps to protect your data.  There is no direct benefit to you to take part in this study. What we learn from this study may help other people in the future.  We will protect your data by storing it in a secure system. We will not store your name with the data.”   - “Do you have any questions?”   “Once we complete these interviews I will be conducting a group interview where you and several other people would see an early version of the tool to help people make decisions about COVID testing. Can I contact you by email at that time to see if you may be interested in that?”  <Researcher can mention there is a compensation for their time – a $75 gift card for 90-120 minutes>  < If participant agrees to be contacted, researcher will obtain preferred contact method either email or phone (text or call)> |

| Permission to record | <Researcher will read this verbatim>  “I would like to record our discussion and then will then send the recordings to be transcribed. This is so that I have your responses in your own words. I will destroy the recording at the end of the study.”  “I will not include any information that could identify you with any of your responses in a report or publication.” |
| --- | --- |
| Start two audio recording devices | “May I start recording now?”  <If yes, start both recording devices and state>  ”Ok, I have started recording.” |

| **CIT Semi-structured Interview:**  Interviewer is to use the questions below in quotations plus context specific probing follow-on questions | |  |
| --- | --- | --- |
| Step 1.  CIT- Establish the Aim | “Let’s begin by having you think for a moment about a recent time when you had to make a decision about testing for COVID” |  |
| Step 2.  CIT – Critical Incident | <Researcher will collect 2 critical incidents if possible by collecting each incident fully and separately. If 2 are not possible it is acceptable for the researcher to collect 1 incident to include robust story detail about decision making for COVID testing  1^st^ incident = a time when they decided TO test for COVID  2^nd^ incident = a time when they decided NOT TO test for COVID  Also acceptable  A time when the person was expected to test for COVID  If the person reports no testing ever, no struggles with a testing decision, never having been asked to test, then exclude from the study.>  “What’s your general approach to COVID testing?”  “I would like you to think about a time when you decided TO test for COVID and a time when you decided NOT TO test for COVID. I would like you to describe these events in detail like a story. I will ask you follow up questions about some of the details as you tell the story”  “Let’s start with a recent time when you decided to get tested for COVID”  “Please tell me about your decision in detail”  <Alternatively>  “Was there a time when you were expected to test? Please describe that in detail”  <Researcher pause here and let person tell the story>  “What was involved in your decision here?  “What was important?  “What were you thinking about?”  <REQUIRED: Next, the researcher probes about timelines and decision points related to the timeline>  “Can you now describe a timeline of events around this decision?”  “For example: “  “What was happening before you were faced with that decision?”  “What was happening to you and those around you during the decision?”  “What happened after the decision?”  “I have some paper and a pen here and would like you to draw the timeline while we discuss it.”  <researcher offers paper and pen/pencil or opens digital timeline sheet on Zoom>  “Go ahead and mark the key decision points on this timeline and let’s discuss those”  <REQUIRED: For each decision point on the timeline, the researcher asks these 3 questions>  “What were your goals?  “What information did you need? Or “What information would have been helpful to you in making a decision?”  “What did you expect to happen?  <Researcher continues to probe the timeline about decision points along the timeline described or drawn. Ask participant to draw decision points on the timeline and describe those as they do it>  *Additional information to clarify incident:*  “What were the reasons for the decision that you made?”  “Where did you test” or Where would you test if you had tested?”  “Why did you choose that location to test?”  <After completing first critical incident interview fully, researcher will start this process over and complete second critical incident>  <Research go back to Step 2 and begin 2^nd^ incident interview>  *Additional general questions*  “Do you trust COVID testing results – either positive or negative?”  “What if you tested positive for COVID?” “Who would you tell?”  “What would be your main concerns?  “Would you test if…  “Exposed to someone who has COVID?”  “You had symptoms of COVID?”  “Traveling?”  “Visiting relatives”  “Going to an event with a lot of people close together”  “What circumstances would prompt you to consider testing?”  “How would you go about being tested for COVID?”  “Would you use a home-based test for COVID?”  “If you wanted to test from home, how would you do that?”  “What would you see as the main benefits of testing?’  “What concerns would you have about getting a COVID test?”  “What are/would be your concerns when using a home-based test”?  “What if you could request free at-home test kits on a phone app when needed?”  <Researcher continues to probe using based on responses and CIT technique – What happened next? What did you do next? What were you thinking? How did that effect you? Etc. >  “Do you have any final comments to share with me before we finish this part?” |  |
| Stop/End | “Thank you, I’m now going to stop the recording”  <Turn the recording off before obtaining email address>  “Ok the recording has stopped now” |  |
| **Demographic Data Collection** | |  |
| Step 1.  Demographic data sheet | <Capture the demographic data on a separate sheet with a participant # assigned to it. Collect this data after the interview to minimize priming. >  “Next, I would like you to answer the following questions”  <Researcher gives the person the demographic data sheet to review and answer or show demographic sheet on Zoom to answer verbally – do not alter this NIH mandated format for #s 1-4: >   1. What is your age in years? 2. What is your gender?    1. Male    2. Female    3. Other________ 3. Please indicate all that apply    1. American Indian    2. White    3. Black    4. Asian    5. Hawaiian    6. Other    7. Unknown 4. Ethnicity    1. Hispanic or Latino    2. Not Hispanic or Latino    3. Unknown 5. Highest level of schooling/education? 6. What is your home zip code? 7. What email address would you like me to send your $50 gift card to? |  |
| **Wrap-up** | | |
| Close the interview | “Thank you for your time today. This has been very helpful and your input is very valuable and I really appreciate your participation.”  “I will email your gift card this week and then you will receive it shortly after that.” | |
